# Moral Distress and Pediatric Palliative Care

**DOI:** 10.3390/children11070751

**Published:** 2024-06-21

**Authors:** Sunny Jeong, Angela Knackstedt, Jennifer S. Linebarger, Brian S. Carter

**Affiliations:** 1 Bioethics Center, Children’s Mercy-Kansas City, Kansas City, MO 64108, USA; sjeong@cmh.edu (S.J.); aknacksted@cmh.edu (A.K.); 2 Department of Nursing, Office of Equity and Diversity, Children’s Mercy-Kansas City, Kansas City, MO 64108, USA; 3 Division of Pediatric Palliative Care, Department of Pediatrics, Children’s Mercy-Kansas City, University of Missouri-Kansas City School of Medicine, Kansas City, MO 64108, USA; jslinebarger@cmh.edu; 4 Departments of Pediatrics and Medical Humanities & Bioethics, University of Missouri-Kansas City School of Medicine, Kansas City, MO 64108, USA

**Keywords:** pediatric palliative care, moral distress, communication, consultation, ethics

## Abstract

Moral distress is a complex phenomenon whereby a person feels tension, constraint, or conflict with an action or circumstance because it goes against their individual or the perceived collective (e.g., community, organizational, or professional association’s) moral stance. In pediatric healthcare settings, managing and mitigating feelings of moral distress can be particularly difficult to navigate through because of the intricate dynamics between the pediatric patient, parent and/or legal guardians, and clinicians. The proactive integration of an experienced pediatric palliative care (PPC) team can be an appropriate step toward reducing clinicians feeling overwhelmed by various case-specific and team management issues that contribute to the development of moral distress among healthcare professionals. Based on our experiences in a free-standing, quaternary pediatric hospital, the involvement of PPC can help reframe the approach to challenging situations, enhance communication, and provide guidance to the care team, patients, and families. Moreover, PPC teams can benefit other multidisciplinary team members through education on respecting the plurality of values of diverse families and patients and consideration of ethical implications during morally challenging situations.

## 1. Introduction

Moral distress is a complex phenomenon whereby a person feels tension, constraint, or conflict with an action or circumstance because it goes against their individual or the perceived collective (e.g., community, organizational, or professional association’s) moral stance. Moral distress occurs across all healthcare professions and settings, particularly nurses and those working in intensive care units [1,2]. Moral distress can have tumultuous long-term effects on healthcare professionals such as job dissatisfaction, burnout, resignation, and more [1,3]. Amplified by the events of the COVID-19 pandemic, moral distress has increasingly been a topic of concern for many healthcare professionals [3,4,5]. With the recurrence of episodes of moral distress over time there may be an accrual of distress leading to what has been described as a moral residue, which in turn, can affect a clinician’s physical and mental well-being and the quality of care that they provide to patients and their families [6]. To efficiently attend to an individual or collective experience of moral distress, it is necessary to understand how it has been manifested. 

Identifying the sources of clinicians’ feelings of moral distress can potentially help them reassess challenging situations and rebuild their approaches to providing high-quality care with confidence and integrity. Moral distress is particularly heightened in pediatric clinicians who work in critical or intensive care units [3,7]. Crowe et al., denote two primary risk factors that lead to moral distress: (1) “the inability to prepare for the emotional demands and end-of-life care involved in their roles”, and (2) “the acute and chronic psychological distress as a consistent feature” [8]. Other common causes of moral distress include resource constraints, disagreement on treatment plans between the patient-parent-health care professional(s) triadic relationship, tension on whether a care plan aligns with the patient’s or family’s values or feeling restricted on advocating for the patient [9,10]. Moral distress may also represent a gap in knowledge in a trainee who expects more of themself than is reasonable at a given point of training, or it may develop when clinicians feel incompetent for not being able to perform their perceived professional duties and obligations, especially as a child’s medical status declines [3,9,10,11].

Pediatric clinicians often encounter patients and their caregivers during a time of great upheaval in their lives as they deal with uncertainty, fear, and potential loss. Beyond caring for their physical body, true holistic well-being involves caring for their moral being and emotions. To navigate patients’ and families’ complex moral terrains, clinicians must cultivate their ability to elicit (through empathic inquiry) and resonate with patients’ and families’ narratives—their stories—and the goals, values, and emotions attached to them. This will help foster collaborative decision-making, produce thoughtful care plans in difficult circumstances, and mitigate feelings of moral distress. Pediatric palliative care (PPC) clinicians’ training and certification focuses on these components of patient care [12]. It is for these reasons that the integration of PPC clinicians as members of care teams has exciting potential to help other clinicians strengthen their moral resilience and reduce the frequency and intensity of moral distress [1,2]. This will subsequently improve the healthcare experience for the pediatric patient and family by providing effective and meaningful treatment plans, especially when the child has a serious or life-threatening illness.

Patient- and family-centered care is a foundation for most pediatric institutions but may not be complete without the integration of PPC. Using the example of a free-standing pediatric hospital’s robust PPC team, we will discuss how early referrals to integrate PPC in the care of patients with serious illnesses may help mitigate or address the care team’s feelings of moral distress. There are two key paths to PPC referrals (1) a clinical scenario that prompts a particular order set (e.g., for tracheostomy evaluation), and (2) socialized diagnoses or situations that motivate the healthcare team to consult PPC clinicians. The second type of referral occurs when the “primary” clinician or medical team for the patient identifies a diagnosis or circumstance for which PPC may be beneficial such as serious fetal anomalies, concerning oncology prognoses, or conditions with complex medical decision-making on the horizon. While these prompts and socialized norms take time to develop and win agreement across units of care (not always symmetrically), in our experience, having PPC support has strengthened resilience to reduce moral distress and enhanced clinicians’ understanding of morally challenging situations.

## 2. Re-Framing What Pediatric Palliative Care Does

Pediatric palliative care specialists and teams focus on patients with serious, life-threatening, or life-limiting, illnesses. They accompany the patient, family, and healthcare team in navigating the physical, psychological, and social complexities of serious illnesses. The World Health Organization recommends palliative care begin when a child is diagnosed with such a condition, allowing support in adjusting to the illness, its impact on their quality of life, and the ongoing assessment of patient and family values and goals of care throughout the illness trajectory and across care settings. Unfortunately, PPC is often misunderstood and considered to be synonymous with end-of-life or hospice care [13,14,15]. When PPC is believed to only relate to death and dying, healthcare professionals may have internal turmoil and moral distress regarding when to involve the PPC team. Thus, it is imperative to re-frame the role and purpose of PPC teams as it can shift people’s perception of their critical presence.

Despite technological advancements and the development of medical knowledge over the years, clinicians and trainees alike (i.e., residents, specialty fellows, nursing students, medical students, etc.) still categorize success as prolonging life and equate death with failure [13]. This binary view prevents clinicians from practicing with patient-and family-centered care as a foundation because the individual or care team focuses on curing, not healing. Although the two concepts are often used interchangeably, they are distinct. *Curing* is often associated with the disease-oriented model of medicine, where clinicians are actively trying to *fix* or eliminate a certain disease or disease-related symptoms [16]. In contrast, *healing* focuses on the sick person as a whole and attends to their quality of life and wellness. Healing is subjective to the individual person and encompasses their beliefs and expectations of what it means to live well and with dignity [16].

For clinicians anchored in the framework of cure, the involvement of PPC may insinuate that they are “giving up” or failing to fix the child. Such a perception may be due to a clinician’s implicit bias, which should be further explored. While PPC clinicians have cases where transparent discussion of end-of-life or hospice care is warranted, their purpose is to provide holistic and comprehensive care to children with a life-threatening or serious diagnosis [15]. PPC clinicians are trained to help with a myriad of challenging situations such as clarifying the patient and family’s goals of care, providing advance care planning, and facilitating dialogue about the role of life-sustaining therapies or interventions. Hence, an underlying implicit bias against PPC can result in delayed referrals, impeding the ability of the PPC team to build relationships and explore values and goals before a moment of crisis. This in turn may leave the patient and family with suboptimal benefits offered too late as the child’s medical condition can rapidly deteriorate.

PPC clinicians often bear witness to moral distress in care teams ranging from differences in opinion about which treatment plan is considered “best” by a given clinician, or differences in the benefit/burden assessment for the patient, to scenarios in which patients and families decide to forego difficult, complex treatment plans altogether. Many times, the inclusion of PPC clinicians can be helpful to the primary healthcare team members by supporting them in managing these complex situations. In Leland & Wocial’s revisit of *Residents Still Struggle When Children Die*, the authors state that the death of a pediatric patient, whether the circumstances were sudden or expected, invokes feelings of grief, frustration, and more [10,13]. The early involvement of PPC—ideally from the point of making a serious diagnosis forward, or early in a child’s hospitalization—is a proactive measure to reduce the accumulation of moral distress amongst pediatric clinicians. While the focus of PPC may be the patient and family, they have an integral role in supporting the healthcare team members involved.

Having a PPC referral for pre-determined diagnoses or when there is a significant decline in a patient’s baseline can benefit the patient, family, and healthcare team. PPC clinicians can help explore and prioritize what quality of life means for the patient and family, address symptoms, articulate the hopes and worries, and form collaborative, therapeutic partnerships between the patient-parent-provider [17]. Working alongside primary care teams, PPC’s involvement can identify opportunities to re-focus the care to improve the child’s quality of life throughout the progression of the patient’s illness experience across care settings and may call attention to the balance of treatment benefits and burdens. At times this could look like finding an option that promotes responsible and thoughtful considerations of withdrawing a no longer appropriate treatment or providing comfort and supportive care [13]. Incorporating PPC principles in the care team can help clinicians, parents, and patients prepare for unpredictable outcomes and provide opportunities for reflection.

In our institution, our well-established PPC team provides critical resources to patients, families, and staff to facilitate productive discourse and mitigate feelings of moral distress. Our PPC team intentionally endeavors to educate healthcare team members individually as needed and willfully joins clinical team rounds and care conferences to do so. These actions serve to help reframe the perception of PPC solely being an end-of-life-focused resource. This educational role can also be helpful in working with patients and parents to facilitate their own understanding of the disease condition, current status, trajectory, and anticipated outcomes [18]. In many institutions, there are pre-determined diagnoses that carry grave prognoses for which it has become socialized and acceptable to consult PPC. In such instances, it is possible for key stakeholders to better appreciate early on what PPC can offer. Patients, families, and care teams working together are then able to form collaborative partnerships with each other and become a cohesive unit for the patient and family’s benefit. Clinicians may also gain insights to how obtaining a PPC consultation can enhance their understanding of critical decision-making, such as why families and patients may decide to make decisions to continue life-sustaining efforts even if accompanied by burdens, or alternatively, to limit further cure-oriented and life-extending care. Such decisions may require exploration and validation through the lens of different value systems, priorities, or choice architecture than what the primary clinical team is used to. The common language in the PPC world includes informing parents of “what other loving families in similar situations have chosen to do”. Together, the primary clinical team, the patient, and the family will experience a spectrum of emotions and considerations—all of which are valid. The PPC team that is involved early and contacted at times of serious or unfavorable changes in the child’s prognosis can be expected to provide high-quality care until there is improved stability, remission, or transition to an adult care setting. This proactive measure of knowing when and how to involve the PPC team has reduced potential tension and feelings of moral distress.

## 3. Pediatric Palliative Care as an Aid for Communication

Poor communication can be a significant contributor to moral distress. Misinformation and disinformation can lead to misunderstandings, tension, and unmet patient and parental expectations—further adding to feelings of moral distress.

Early PPC referral and involvement can facilitate timely discussions about patients’ and parents’ values and goals of care underlying the treatment decisions. Longitudinal PPC involvement ensures goals of care discussions are revisited, as goals may change over time [19]. Healthcare teams experiencing moral distress may find support in having discussions about patient-and-family preferences, debriefs on what factors contributed to their feelings of distress, and even identifying systemic injustices that had an impact on the outcome [20]. PPC clinicians can lend their expertise as active listeners and mediators to ensure key stakeholders’ voices are being heard, valued, and supported [21].

To provide safe and comprehensive care, high-quality communication between the patient, family, and healthcare teams is critical [22]. However, when clinicians are not comfortable with, disagree with, or are not satisfied with certain treatment plans or prior conversations, the quality of communication can diminish. For example, in high-stress situations, crucial conversations that address the provision of aggressive or unpredictable treatments are warranted should an urgent or emergent situation occur [10]. In such situations, where complex decisions must be made, tremendous stress can accrue from an attendant emotional burden on the healthcare team’s members [10]. Not all pediatric clinicians have formal training on how to have difficult conversations with patients and families [8,22]. The early engagement of PPC clinicians can aid in creating a strong foundation of open and receptive communication that promotes shared decision-making [22].

Over the years, shared decision-making has been adapted into pediatric practice in North America [23]. However, during complex situations where time and resources may be limited, clinicians may find it challenging to have difficult conversations with patients, families, and other members of the interprofessional team. The guidance and support provided by the PPC team can help the primary team feel confident in the decisions that are being made with the patient and family. An exemplary practice of shared decision-making may well include consulting with PPC clinicians who can help the primary team ensure that when compromises in outcomes or goals are made due to clinical necessities, they are appropriate [23]. Although members of the primary medical team may have training in promoting shared decision-making, they may not have a holistic overview of what matters most to the patient and family. PPC clinicians are trained to elicit information from both the patient and family regarding their goals and have comprehensive knowledge about what may be important goals (i.e., reaching a birthday or seeing a sibling graduate) of the patient and family in their healthcare journey [23]. The intimate relationship PPC teams form with patients and families ensures that relevant clinical information is being considered by the interprofessional team, while patients’ and families’ values and preferences are honored, bringing back some control over their decisions.

We have recognized that the involvement of the PPC team helps reduce the frequency and intensity of feelings of moral distress because it initiates the beginning of a longitudinal relationship, which is also beneficial for long-term communication and continuity of care [1,2]. When the PPC team receives consult requests for a particular diagnosis or when there is a change in a patient’s baseline, it will follow the patient across hospital and clinic environments and encounters. A longitudinal relationship with PPC team means that a group of clinicians holds knowledge about the patient’s course of care and understanding of family values and dynamics which can allow the primary clinical care team to form rapport faster.

The involvement of PPC fosters the development of trust and transparency, enhancing otherwise formidable communication. PPC clinicians have advanced training to better elicit the reasoning behind families’ choices. With these strong mediators, a family’s rationale of what they consider to be in the best interest of their child can be better relayed to the primary healthcare team and negotiated with the collaborative partnerships of the interprofessional team [20,21]. In addition, through the support of PPC clinicians, the mediation process can be a space where potential alternative courses of care plans and expected outcomes can be readily communicated [21]. It is common for clinicians to experience moral distress when they disagree with the patient and family, but the PPC team members can speak about the history and reasoning of each unique patient and family, mitigating feelings of moral distress.

Difficult and emotional conversations with patients, families, and healthcare team members must be executed sensitively. PPC clinicians can help the primary team in facilitating effective and receptive communication as they help ensure information is being processed and understood [23].

## 4. Understanding Pediatric Palliative Care’s “Frame of Reference” and toward Plurality

Holistic patient-and-family-centered care requires the collaborative partnership and efforts of an interprofessional team composed of physicians, nurses, social workers, and others. Different professionals offer critical perspectives, expertise, and methodologies in approaching clinical care; thus, individuals from diverse professional backgrounds are encouraged to engage, learn together, and practice to deliver the highest quality of care. However, each profession and subspeciality within a field carries different normative priorities and core foundations that direct the way they perceive patient needs and provide care. Some specialists are more focused on the clinical status of the patient, often within an organ system or disease-based manner; whereas the frame of reference used by the PPC clinicians tends to be holistic—focusing on the entirety of the patient as a unique person and a member of a family unit.

Moral distress has been rooted in the fact that clinicians feel constrained from acting in what they believe to be the most ethically sound manner; thus, going against their moral agency [24]. In Fiester’s recent address of moral distress in the *Journal of Clinical Ethics*, she proposes a new intervention to teach healthcare professionals the value of pluralism. This intervention focuses on helping clinicians shift their “frame of reference” to distinguish between “unjustified and justified constraints” [24]. Often there may be competing opinions, thought processes, and values that contribute to feelings of moral distress. This incompatibility creates a barrier to support and the highest quality of care for patients. For example, parents may have a different assessment of what is in the best interest of the child compared to clinicians. In the pediatric healthcare paradigm, both families and healthcare teams have the goal to attend to the best interest of the patient whereby all parties involved are considering what action would promote the most reasonably sufficient, or maximum, “good” for the child [25]. However, families may misuse their privilege and promote their own values over the patient’s. On the other hand, clinicians may focus too closely on the clinical aspects of care and not consider the relevant contextual and external influences on the particular child’s care. In such cases, PPC clinicians may prove helpful in deriving an optimal course of care that is considerate of all parties’ perspectives. No well-intended assessment of the best interest is inherently wrong or incorrect but due to each party’s contrasting moral positions, priorities, cultural or religious influences, and value systems, a resultant dissonance may create the perception of some violation or wrongdoing [24]. Therefore, the practice and acknowledgment of robust pluralism are both relevant and necessary in pediatrics generally and palliative care specifically. Robust pluralism acknowledges other moral theories, commitments, insights, and perspectives to approach moral deliberation. Through continuous critical analysis and appraisal of various accounts’ strengths and limitations to moral circumstances, robust pluralism elevates the archetypal moral pluralism. The ever-evolving process of moral deliberation in medicine, palliative care, and bioethics requires the acknowledgment of various moral contributions, and the open-mindedness that robust pluralism brings.

PPC teams can help shift this mentality or “frame of reference” by grounding parents, patients, and clinicians in the understanding that plurality and non-uniformity of value systems are the backdrops of our lives in a broad, diverse, and inclusive society. Privileging and imposing certain values and beliefs can be morally and ethically fraught; thus, contributing to feelings of moral distress. PPC teams may be uniquely positioned to promote the quality of life for critically or seriously ill children and their families, while ensuring the goals of care and their collective *goods* can be prioritized. The burdens of moral distress that arise from the constraints of one’s moral agency can be detrimental to the practice of care.

Fiester’s call to “think differently” rather than to “act differently” is a sagacious step toward mitigating feelings of moral distress [24]. Our PPC teams’ holistic approach has helped other disciplines and subspecialties understand the importance of uplifting the diversity and plurality of values. This practice allows clinicians to be attuned and reflective of themselves, and guides interprofessional teams to be cognizant about *what* they communicate to patients and families and *how* they communicate the information. 

## 5. Consideration of Ethics and Pediatric Palliative Care

Another consideration of how PPC teams can help mitigate feelings of moral distress is by utilizing their skills in bioethics. In many pediatric institutions, PPC providers also serve on ethics committees and contribute further as ethics consultants [20]. Thus, many PPC providers have ethics education, training, and/or experiences, which make them equipped to navigate through morally complex and challenging issues. This ethics background allows PPC providers to consider bioethical inquiries that traverse strict clinical boundaries. Supplementing their rationale and reasoning on challenging cases with ethics or moral frameworks, PPC providers may help other healthcare team members understand important safeguards that need to be considered for the respective patients and families. Additionally, ethics-trained PPC providers may reduce feelings of moral distress by grounding healthcare team members in the reality of patients’ and families’ unique narratives, value systems, and future implications of the decisions made.

If a PPC provider is actively involved in the care of the patient that requires ethical insight, it is recommended that a formal ethics consultation request is made to be assessed by an objective ethics consultant [20]. Nevertheless, a PPC clinician who is trained in ethics can also help team members facing a challenging case to focus on its morally significant components. During situations that provoke feelings of moral distress, clinicians may want immediate solutions to “fix” or “problem-solve” the issue at hand. However, the decisions that patients and families make may not align with their thoughts or opinions. Therefore, PPC providers can help team members have a better understanding of patients’ and families’ decisions by utilizing various principles, moral theories, and values-based approaches used in bioethics. This can categorize and universalize moral judgments while serving as a model to systematically assess moral circumstances.

## 6. A Future towards Moral Distress Education

In a recent *Hastings Center Report*, Kim et al. promoted understanding the term “agent regret” as a factor of moral distress [26]. Agent regret encompasses the idea that clinicians who encounter feelings of moral distress should not feel guilt. Feeling guilt insinuates that there was a certain failure to meet expectations and can amplify feelings of powerlessness in an institutional or clinical capacity [26]. Agent-regret characterizes there being a “desire to make amends”, even if it is simply an acknowledgment of processing feelings of moral distress [26]. However, novice clinicians and trainees may be hesitant or unaware of how to do so appropriately. Over the years, the ethical and clinical complexities behind experiences that contribute to feelings of moral distress have revealed that clinicians feel personally or professionally constrained. This tension created by some form of action, or in some cases, inaction, causes dissonance with what they perceive to be their clinical duty and responsibility. Unfortunately, moral distress will be an ongoing topic that cannot be eliminated as clinicians will encounter everyday ethical challenges. Therefore, to lessen or ameliorate feelings of moral distress, clinicians and trainees must receive education on how to develop appropriate coping mechanisms with the aid of PPC teams.

In most studies, moral distress is assessed and evaluated retrospectively and at a single point in time, such as when clinicians are asked to fill out a questionnaire or survey. However, coping with moral distress varies for every clinician and may be dependent on the day-to-day clinical status of their patients [27]. Furthermore, clinicians often find it difficult to navigate through these feelings of moral distress and the myriad emotions they bring because it is a *process* that requires time and appropriate resources to cope. When clinicians encounter seemingly negative experiences that punctuate feelings of sadness, regret, and stress, without appropriate coping skills, it can disconcertingly imprint onto clinicians’ lives and practice. Moreover, when there is great uncertainty in the prognosis of a critically ill pediatric patient, clinicians may have difficulty doing “too much” or “too little”. PPC teams can reorient the goals of care, help clinicians understand the subjectivity of the patient’s pain and suffering, improve knowledge of potential outcomes, and offer resources to appropriately deal with moral distress [27]. In collaboration with PPC clinicians and ethics colleagues, they can help build a strong foundation for moral distress education so that novice clinicians and trainees have a better understanding of the evolving health trajectory of patients, while also practicing timely and appropriate patient- and family-centered care.

Over the past few years with the impact of the COVID-19 pandemic, it has been noted that many trainees have developed poor coping mechanisms to navigate feelings of moral distress and, for some, a growing inability to empathize during ethically challenging situations. For example, in Prentice et al.’s study, it was revealed that novice NICU physicians in their first year have greater experience in their frequency and intensity of moral distress than physicians who have worked for over fifteen years [27]. Learning suitable coping strategies by having formal and informal conversations with PPC team members, along with clinical colleagues, can help navigate through difficult ethically complex situations [28]. Without these conversations, novice clinicians or trainees may start to feel a sense of powerlessness, have complete emotional detachment from patients and families, and even experience cynicism [28]. Fostering appropriate coping habits early on can protect these clinicians from burnout and exhaustion during morally distressing scenarios.

In pediatric intensive care units (PICU) or neonatal intensive care units (NICU), many morally distressing situations arise that challenge the development of clinicians’ personal, moral, and professional integrity. These can have tumultuous effects on any clinician and impact the delivery of patient care [27]. Having appropriate education and training with PPC clinicians can promote an understanding of the different facets of integrity via ethics consultations, unit debriefs, or formal PPC conversations [27,29]. When pediatric clinicians care for critically or seriously ill patients and their families, they face the unique challenge of avoiding the antiquated practice of paternalism, while trying to respect parental authority. Clinicians can learn from PPC clinicians how to uphold their professional duty and obligation to guide parents along a stressful journey, without instigating potential future harms [30]. Despite sufficient clinical training, some clinicians have yet to experience situations whereby patients and families approaching the end-of-life may see their medical judgments as fallible [27]. PPC clinicians can help cultivate moral attunement and awareness by teaching trainees how to express insight, ask meaningful questions of patients and families, consider alternative and diverse perspectives, and address ethical challenges [9,27]. These developments can help ensure clinician resilience while ameliorating feelings of moral distress.

Across the world, there is considerable variability in pediatric palliative care access, practices, and beliefs which are influenced by the local practice patterns of healthcare systems, institutions, individual palliative care teams, and even individuals [31]. Nevertheless, the PPC team, in addition to providing fundamental resources to all clinicians, allows trainees from diverse disciplines to shadow and learn from the team. This engagement from the early stages of training provides an opportunity on how patient cases are approached, how patients’ and families’ values are taken into consideration, specific to unique situations, and how details of a case are discussed within the team. It is beneficial for novice clinicians and trainees to assess their role in difficult situations and have the appropriate approach to accurately interpret and navigate challenging situations.

## 7. Conclusions

The proactive integration of PPC can be an appropriate step to minimize the overwhelming feelings and impact of moral distress upon healthcare professionals. When clinicians attend and care for a child, there are many situations when the ends of the care that is hoped for cannot be met, or perhaps only come with reluctance from caregivers and family. However, in these moments, when healthcare professionals of all disciplines cannot attain a cure, and healing seems a distant oasis, despite the provision of optimal care, they can provide advocacy through the involvement of PPC. Based on our experience, the involvement of PPC can help reframe the approach to challenging situations and in so doing enhance communication and provide guidance to the care team, patients, and families. Additionally, our PPC team members are invaluable assets as they ground and educate care teams to consider the plurality of values of diverse families and patients and their ethical implications during morally challenging situations. Thus, robust PPC teams can prove to be foundational in helping healthcare teams navigate through morally complex situations and mitigate feelings of helplessness and the often-attendant moral distress.

## Data Availability

Data sharing not applicable. No new data were created or analyzed in this project.
